# Eosinophilic gastroduodenitis: a pediatric perspective

**DOI:** 10.1186/s13052-025-02115-3

**Published:** 2025-09-29

**Authors:** Lucia Caminiti, Stefania Arasi, Simona Barni, Riccardo Castagnoli, Mariannita Gelsomino, Mattia Giovannini, Angela Klain, Lucia Liotti, Carla Mastrorilli, Francesca Mori, Luca Pecoraro, Francesca Saretta, Michele Miraglia Del Giudice, Elio Novembre

**Affiliations:** 1 Department of Human Pathology in Adult and Development Age “Gaetano Barresi”, Allergy Unit, Department of Pediatrics, AOU Policlinico Gaetano Martino, Messina, 98124 Italy; 2 Translational Research in Paediatric Specialities Area, Division of Allergy, Bambino Gesù Children’s Hospital, IRCCS, Piazza Sant’Onofrio 4, Rome, 00165 Italy; 3 Allergy Unit, Meyer Children’s Hospital IRCCS, Florence, 50139 Italy; 4 Department of Clinical, Surgical, Diagnostic and Pediatric Sciences, University of Pavia, Pavia, 27100 Italy; 5 Pediatric Clinic, Fondazione IRCCS Policlinico San Matteo, Pavia, 27100 Italy; 6 Department of Life Sciences and Public Health, Pediatric Allergy Unit, University Foundation Policlinico Gemelli IRCCS, Catholic University of the Sacred Heart, Rome, 00168 Italy; 7 Department of Health Sciences, University of Florence, Florence, 50139 Italy; 8 Department of Woman, Child and General and Specialized Surgery, University of Campania Luigi Vanvitelli, Naples, 80138 Italy; 9 Pediatric Unit, Department of Mother and Child Health, Salesi Children’s Hospital, Ancona, 60123 Italy; 10 Pediatric Hospital Giovanni XXIII, Pediatric and Emergency Department, AOU Policlinic of Bari, Bari, 70126 Italy; 11 Pediatric Unit, Department of Surgical Sciences, Dentistry, Gynecology and Pediatrics, University of Verona, Verona, 37126 Italy; 12 Pediatric Department, Latisana-Palmanova Hospital, Azienda Sanitaria Universitaria Friuli Centrale, Udine, 33100 Italy

**Keywords:** Eosinophilic gastroduodenitis, Non IgE-mediated food allergy, Food allergy, Children, Biologicals

## Abstract

Eosinophilic gastroduodenitis (EoGD) is a group of chronic inflammatory disorders characterized by an increased presence of eosinophils in the stomach and duodenum. Although relatively rare, it is an increasingly recognized form of eosinophilic gastrointestinal diseases (EGIDs) with significant clinical implications, particularly in pediatric patients. Despite advancements in diagnostic tools, considerable delays in diagnosis persist due to the nonspecific nature of clinical manifestations, which often resemble more common gastrointestinal disorders. Early referral to a gastroenterologist is critical, especially when patients present with persistent gastrointestinal signs and symptoms that do not improve with treatment for common conditions like irritable bowel syndrome. Food allergens and microbiota may play a role in the pathogenesis of this disease. Management typically involves a combination of dietary modifications, pharmacological treatments such as corticosteroids, and, in some cases, biologic agents targeting eosinophilic inflammation. However, long-term outcomes remain variable, and data on prognosis are still limited, underscoring the need for further research. Future studies should focus on validating new diagnostic markers and therapeutic strategies, better understanding long-term outcomes, and developing personalized treatment plans. A multidisciplinary approach, incorporating the expertise of gastroenterologists, allergists, dietitians, and surgeons, is crucial to ensure optimal patient care and management.

## Introduction

Eosinophilic gastrointestinal diseases (EGIDs) are a group of chronic inflammatory disorders characterized by an elevated presence of eosinophils in the gastrointestinal (GI) tract, without an identifiable cause for the eosinophilia [1]. These diseases are classified based on the location of eosinophilic infiltration, which can occur anywhere along the GI tract, from the esophagus to the colon [2]. The most well-defined of these conditions is eosinophilic esophagitis (EoE), established by international diagnostic guidelines [3, 4]. However, in recent years, increasing attention has been directed toward non-EoE EGIDs, which include eosinophilic gastritis (EoG), eosinophilic enteritis (EoN)—recently subdivided into eosinophilic duodenitis (EoD), jejunitis (EoJ), and ileitis (EoI)—and eosinophilic colitis (EoC) [5, 6].

This narrative review focuses particularly on pediatric eosinophilic gastritis (EoG) and eosinophilic duodenitis (EoD) (notably, hereafter referred together with the acronym EoGD for simplicity when both entities are meant), characterized by an increased presence of eosinophils in the stomach and duodenum, respectively. The aim is to synthesize current knowledge on the etiology, diagnosis, management, and outcomes of these conditions in children, providing a useful guide for daily practice. A comprehensive search of the published literature was conducted using the PubMed and MEDLINE databases. Key terms included ‘eosinophilic gastrointestinal disorders,’ ‘eosinophilic gastritis,’ ‘eosinophilic duodenitis,’ and ‘eosinophilic gastroduodenitis.’ Only English-language studies were considered, focusing on high-quality evidence.

## Epidemiology

Eosinophilic gastroduodenitis (EoGD) is relatively uncommon, with incidence rates not as well-documented as other EGIDs like EoE. However, studies suggest that the incidence of EoGD is increasing, paralleling the rise in allergic diseases in children [7]. According to Sonnenberg et al. [8], the prevalence of gastric eosinophilia was found in approximately 1.21 patients per 1,000 undergoing biopsies, while duodenal eosinophilia was even rarer, with a prevalence of only 0.03 per 1,000 patients. The study highlighted that patients with eosinophilia in one section of the gastrointestinal tract, such as the esophagus or colon, were at significantly higher risk of developing eosinophilia in the stomach or duodenum [8]. This suggests a potential concordance or overlap of these eosinophilic conditions across different parts of the GI tract, indicating the possibility that eosinophilia may affect even distant segments. While EoGD is less frequently diagnosed compared to esophageal eosinophilia, its prevalence may be underestimated due to a lack of awareness among gastroenterologists and pathologists about the importance of performing multiple biopsies in patients with chronic GI signs and symptoms [8].

Several risk factors can increase the likelihood of developing non-esophageal EGIDs, including EoGD. Jensen et al. [9] found that pregnancy complications, neonatal intensive care unit (NICU) admission, and antibiotic use during infancy were all significantly associated with an increased risk of developing EGIDs, albeit with some methodological limits of the study. Specifically, patients exposed to antibiotics in infancy had more than seven times the risk compared to controls [9]. Environmental factors, such as climate, urban area, and dietary components, are also implicated [10, 11]. There is a strong association between EGIDs, including EoGD, and allergic diseases [12]. In pediatric patients, the majority of those diagnosed with EoGD also present with comorbid allergic conditions such as food allergies, asthma, atopic dermatitis, and allergic rhinitis [12]. Particularly, food allergies play a crucial role in triggering EoGD, with patients often responding to elimination diets or achieving histological remission when food allergens are removed from their diet [12].

## Pathogenesis

Eosinophilic gastroduodenitis (EoGD) is primarily driven by a type 2 (T2) immune response, often triggered by food allergens. Epithelial barrier dysfunction in the stomach and duodenum allows allergens to penetrate the mucosa, initiating a cascade of immune responses that perpetuate local inflammation [13]. This process is characterized by elevated levels of Th2-associated cytokines, including IL-4, IL-5, and IL-13, which play a central role in recruiting eosinophils and activating mast cells in the affected tissues [13, 14]. Once activated, eosinophils release eosinophil-derived neurotoxins (EDN) and eosinophil cationic protein (ECP), which cause tissue damage and exacerbate inflammation [13, 14].

A hallmark of EoGD is the infiltration of eosinophils into the gastric and duodenal tissues, a process further amplified by the presence of type 2 innate lymphoid cells (ILC2s) and mast cells, which contribute to the ongoing allergic inflammation [15]. Additionally, IL-5 promotes eosinophil activation, leading to tissue remodeling and, in severe cases, to mucosal and submucosal fibrosis, contributing to the chronic nature of the disease [15].

While the underlying T2 inflammatory response is common across EGIDs, EoGD exhibits distinct yet overlapping mechanisms compared to other EGIDs [16]. For instance, although eotaxin-3 and IL-13 are key factors in eosinophil recruitment in EoE, CCL11 (eotaxin-1) plays a more significant role in attracting eosinophils to the gastric and duodenal mucosa in EoGD [16]. This indicates that while the broader Th2-driven inflammatory pathway is shared across EGIDs, the molecular players and specific tissue responses can vary depending on the segment of the gastrointestinal tract involved [16].

### The role of microbiota in EoGD

Recent evidence suggests the potential role of the mucosal microbiota in the pathogenesis of EoGD. According to Furuta et al. [17]. , patients with EoG exhibit substantial shifts in their bacterial communities compared to healthy controls. Notably, in healthy individuals, the bacterium *Prevotella* is predominant in the stomach, suggesting it may play a protective or regulatory role in maintaining normal gastric mucosal function. In contrast, in patients with EoG, there is an increased abundance of *Streptococcus*, regardless of disease activity [17]. This shift may be indicative of its association with the inflammatory processes characteristic of EoG [17]. Additionally, patients with active EoG display a significant increase in *Leptotrichia*, a bacterium linked to inflammatory conditions, suggesting its abundance may be associated with disease activity [17]. These alterations in microbial composition may perpetuate inflammation by modulating local immune responses, contributing to tissue damage [17]. The findings underscore the importance of further research to clarify whether microbial shifts are causal or secondary to EoG/EoD, how these shifts influence inflammation and disease progression in EoG/EoD, and whether targeting the microbiota could present a viable therapeutic approach.

## Clinical presentation

Children diagnosed with non-EoE EGIDs often exhibit a range of signs and symptoms that depend on the GI segment involved, the extent of eosinophilic inflammation, and the depth of inflammation through the bowel wall [5]. These clinical manifestations are typically nonspecific and can overlap with other gastrointestinal conditions, making diagnosis challenging. Regarding EoGD, common signs and symptoms include nausea, vomiting, and abdominal pain [14]. Diarrhea is less common and often associated with involvement of the rest of the small intestine and colon. Due to the combination of decreased appetite, frequent vomiting, and malabsorption associated with EoGD, affected children often experience weight loss and failure to thrive [5, 14]. This can be particularly concerning in growing children, as adequate nutrition is critical for normal growth and development [5, 14].

Classically, EGIDs were classified into three variants: mucosal, muscular, and sub-serosal involvement, with the mucosal variant being the most common [18]. The muscular type is notably linked to bowel-wall thickening, intussusception, and intestinal and gastric outlet obstructions. Conversely, the serosal form is more often associated with the occurrence of eosinophilic ascites, peritonitis, and intestinal perforation [19, 20]. It is important to highlight that individuals with EoGD who suffer from persistent gastrointestinal signs and symptoms often experience a significant decline in their health-related quality of life (Hr-QoL), manifesting increased psychological stress, impeded social relationships, and physical discomfort [21].

## Diagnosis

Diagnosing EoG and EoD remains a significant challenge due to the nonspecific nature of clinical manifestations and the rarity of these conditions, particularly EoD [8]. Typically, they are more common in children under 5 years of age [22]. Patients often present with signs and symptoms that overlap with more common gastrointestinal disorders, such as irritable bowel syndrome (IBS) or functional dyspepsia, leading to considerable diagnostic delays, especially in younger patients [8, 23]. In a longitudinal cohort study, Chelade et al. [24] reported that adult and adolescent patients with EoGD typically consult an average of 7.2 clinicians before receiving a correct diagnosis, with delays averaging 3.6 years. Common clinical manifestations such as abdominal pain, vomiting, and diarrhea were frequently misattributed to other conditions, while important clinical signs like anemia, peripheral eosinophilia, and malabsorption were often overlooked, further contributing to diagnostic delays [24].

**Fig. 1:**
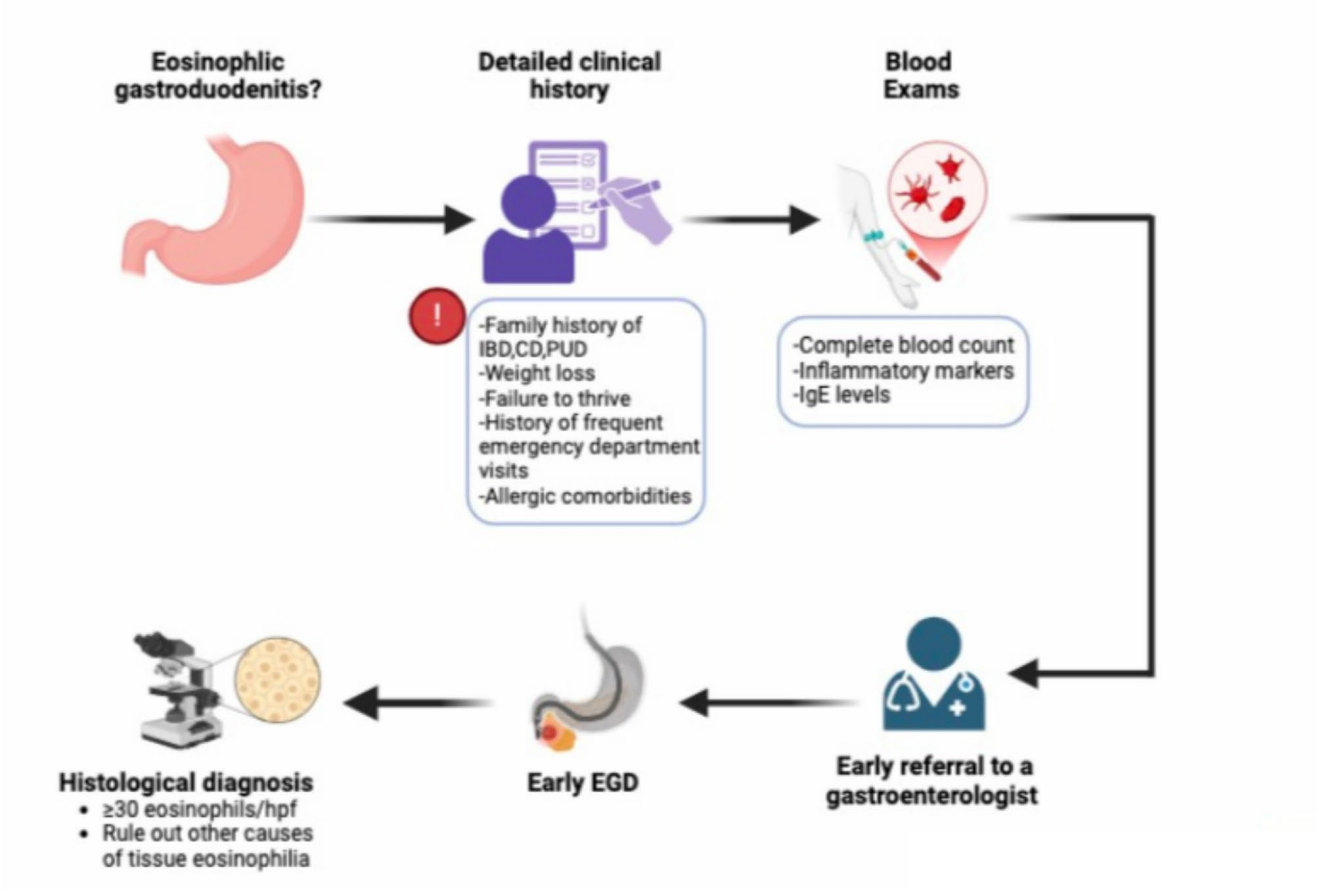
Diagnostic approach to eosinophilic gastroduodenitis

Given the lack of standardized terminology and diagnostic guidelines, a structured diagnostic approach is crucial to help clinicians diagnose EoGD more efficiently and reduce delays. The process begins with a thorough review of clinical manifestations, focusing on warning signs such as weight loss, failure to thrive, or a history of frequent emergency department visits, particularly when signs and symptoms have been misdiagnosed as functional disorders. Potential allergic comorbidities, such as asthma, eczema, or food allergies, should also be considered [24] [Figure 1].

An initial laboratory work-up should include a complete blood count (CBC) to check for anemia and eosinophilia, both of which support the suspicion of EoG/EoD. Specifically, eosinophilia is not always present, as some patients may have a normal eosinophil count while still exhibiting significant eosinophilic infiltration in the gastrointestinal mucosa. There is no universal threshold value, but an eosinophil count > 500/mm³ should raise suspicion of an eosinophilic condition in the presence of persistent gastrointestinal symptoms and/or other atopic manifestations. A count exceeding 1000/mm³ further increases the likelihood of an eosinophilic disorder and should prompt a gastroenterological evaluation. Additional testing for inflammatory markers and IgE levels may provide further insight into underlying inflammation and potential allergic triggers [24].

Early referral to a gastroenterologist is critical, especially when patients present with persistent gastrointestinal signs and symptoms that do not improve with treatment for common conditions like IBS or dyspepsia [24]. In such cases, performing an esophagogastroduodenoscopy (EGD) early, even in the absence of alarm clinical manifestations, is essential, although there is currently no recommendation for specialists to perform an early biopsy for functional disorders [24, 25]. History of EoE, allergic disease and/or peripheral eosinophilia increase clinical suspicion [24]. A validated symptom assessment questionnaire for EoG and EoN has recently been issued for patients > 12 years and is applicable in clinical trial in children with non-EoE EGIDs [5]. Endoscopic findings such as mucosal granularity, nodularity, or erythema should be carefully evaluated and scored using the Eosinophilic Gastritis Endoscopic Reference Score (EGREFS), which is well-associated with histologic findings [26]. During histological evaluation, a diagnostic threshold of ≥ 30 eosinophils per high-power field (hpf) in the stomach and/or duodenum is suggestive [24]. To avoid false negatives, biopsies should be taken from both normal-appearing and abnormal-appearing areas of the stomach and duodenum [26].

Emerging molecular diagnostic tools can provide additional insights into ambiguous or complex cases. For EoG, the EGDP18 panel identifies 18 key genes, including CCL26 (eotaxin-3) and IL13RA2, which can differentiate patients with active disease with high sensitivity and specificity [27]. Similarly, a molecular panel for EoD detects 382 genes associated with duodenal eosinophil levels, effectively distinguishing EoD from duodenal eosinophilia not related to EGIDs [28]. These molecular tools not only assist in early diagnosis but also help monitor disease severity and treatment response [27, 28].

Molecular diagnostics can also help differentiate EoGD from other gastrointestinal conditions such as EoE, celiac disease, inflammatory bowel disease (IBD), and parasitic infections [27, 28]. Once a diagnosis is confirmed, ongoing monitoring is essential to track disease progression, response to treatment, and the potential for recurrence. In some cases, repeat biopsies or molecular diagnostic tests can be used to monitor disease activity, particularly in patients without clinical manifestations who may have residual inflammation [24].

## Management and treatment

**Fig. 2:**
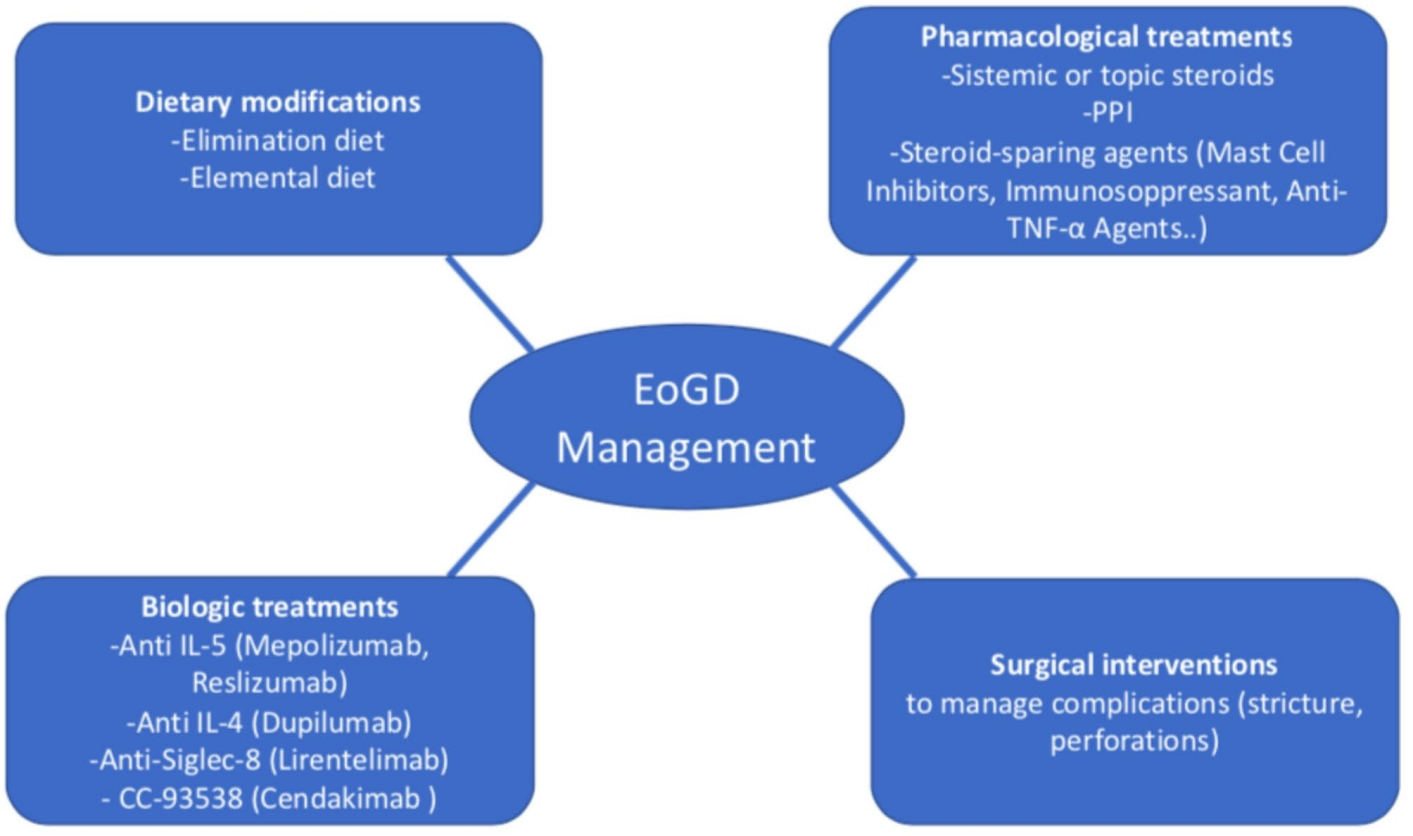
Management of EoGD

The management of EoGD involves a combination of dietary modifications, pharmacological treatments, and, in some cases, surgical interventions [1, 5]. Treatment aims to reduce eosinophilic inflammation, alleviate signs and symptoms, and improve quality of life [1, 5] [Figure 2].

### Dietary modifications

Elimination diets can lead to clinical improvement or remission in some children with EGIDs. However, data on the histological response to these diets remain limited, particularly in pediatric populations [29]. Notably, dietary interventions may influence mucosal microbiota, contributing to signs and symptom relief, although this relationship requires further exploration [17].

Studies indicate that elemental diets can induce clinical remission in approximately 75% of children with eosinophilic gastroenteritis and colitis, though compliance remains a challenge, particularly among adolescents and adults [30, 31]. Although empiric elimination diets are the most commonly used (e.g., the elimination of the 6 most common food antigens such as milk, cereals, egg, soy, seafood, and/or fruits, and the 7-food elimination diet, excluding also red meats), elemental diets tend to be more effective, especially in children with more severe conditions [30, 31].

It is important to note that responses to food elimination often do not correlate with food sensitization identified by skin prick tests (SPT) or serum-specific IgE (sIgE) levels. Patients often are positive for food or aeroallergens tested with a SPT but the value of this test is unclear. At this moment, there is no evidence to support the use of SPT and/or sIgE to manage restriction diets [5].

Different studies compare the efficacy and patient compliance of a milk elimination diet versus a 6-food elimination diet in EoE. Findings suggest that the milk elimination diet may be as effective as the 6-food elimination diet, offering better adherence due to its simplicity [32, 33]. However, it remains unclear whether the selective response to milk elimination can be generalized to all EGIDs.

Nagashima et al. [34] explored a new dietary approach, the Rainbow Elimination Diet (ED), for pediatric patients with EoG and EoD. This diet incorporates an amino-acid-based formula along with hypoallergenic foods such as potatoes, vegetables, and fruits. The study reported that all patients experienced clinical improvement, with enhanced serum protein levels and resolution of clinical manifestations [34]. These findings suggest that carefully tailored elimination diets are both effective and well-tolerated in pediatric patients with EGIDs.

### Pharmacological treatments

Corticosteroids remain a cornerstone in the treatment of EoGD as they effectively reduce inflammation and eosinophil levels in the gastrointestinal tract [5]. They are typically prescribed when dietary modifications alone are insufficient to control clinical manifestations, or in severe cases where rapid relief is necessary [5]. Topical steroids, such as budesonide or fluticasone, are often administered in a swallowed form to target affected areas directly, reducing local inflammation with minimal systemic absorption [35, 36]. This method lowers the risk of systemic side effects. In contrast, systemic corticosteroids, like prednisone or prednisolone, are reserved for short-term use, particularly during acute exacerbations or severe disease, due to their potential for significant side effects​ [35, 36]. Approximately 20% of patients with EoGD develop corticosteroid dependency, highlighting the need for alternative therapies​ [37].

Proton pump inhibitors (PPIs) are also commonly used in EoGD management, particularly for addressing acid-related clinical manifestations and reducing inflammation [5]. However, their role remains controversial, unlike their well-established use in EoE. Gurkan et al. [38] reported that disease control is achieved in roughly 50% of children using a combination of corticosteroids and PPIs, suggesting a role for PPIs in EoGD treatment, though their use should be tailored to the patient’s needs​ [38]. Moreover, Ng et al. (2022) demonstrated the efficacy of PPIs when combined with food elimination diets in achieving histological remission in pediatric patients with EoG, particularly in younger populations, with remission rates as high as 68.8%​ [29].

Steroid-sparing agents are often employed to mitigate the long-term side effects of corticosteroids. These include mast cell inhibitors such as sodium cromoglycate or ketotifen and leukotriene receptor antagonists like montelukast [36]. However, data supporting their effectiveness still needs to be improved. Additionally, medications commonly used in IBD, such as azathioprine, and biologic agents like infliximab and adalimumab, have been tried in severe EoGD cases with varying success [5, 36].

### Monoclonal antibodies

Biologic treatments targeting eosinophil-associated pathways offer promising therapeutic options for EoGD, though clinical outcomes remain variable [36, 39]. Mepolizumab and reslizumab, both targeting IL-5, have shown reductions in eosinophil levels, but clinical improvements have been inconsistent [40]. Benralizumab, an IL-5 receptor alpha (IL-5Rα) antagonist, has shown efficacy in depleting eosinophils through antibody-dependent cell-mediated cytotoxicity (ADCC) [40, 41]. In a recent phase 2 randomized controlled trial, benralizumab significantly reduced eosinophil counts in the blood and gastric tissue of adults and adolescents with EoG [41]. Despite these histologic improvements, changes in patient-reported signs and symptoms, endoscopic findings, and molecular markers of disease were less pronounced. This suggests that eosinophil depletion alone may be insufficient to resolve all features of EoGD, indicating the presence of eosinophil-independent pathogenic mechanisms [41].

Dupilumab, a monoclonal antibody that blocks IL-4 and IL-13 signaling, has shown promise, particularly in EoE [42, 43]. By inhibiting these pathways, dupilumab reduces tissue eosinophilia and promotes clinical improvement [42–44]. Ongoing studies in adolescents and adults aim to further evaluate its efficacy and safety in EoGD (NCT05831176, NCT03678545) [45, 46].

Other biologics, such as lirentelimab (an anti-Siglec-8 antibody), have also been investigated. Lirentelimab effectively reduced gastrointestinal eosinophils and improved clinical manifestations in phase 2 trials, though a phase 3 study (EoDyssey) failed to demonstrate significant improvement despite achieving histologic endpoints in adults and adolescents [47]. Another phase III study is ongoing to evaluate the efficacy of CC-93,538 (cendakimab) in adolescents and adults with EoG and EoD (NCT05214768) [48]. There is insufficient data to recommend any biologic therapy for routine use in non-EoE EGIDs, especially in pediatric populations [36, 39]. Ongoing trials will further clarify their role in disease management.

### Surgical interventions

Surgery is not a first-line treatment for non-EoE EGIDs but is an important option for managing severe complications, such as strictures or perforation [5]. A multidisciplinary approach involving gastroenterologists and surgeons is essential for optimal patient outcomes. Regular follow-up and monitoring are crucial to manage post-surgical complications and adjust ongoing medical therapy as needed [5].

## Prognosis and long-term outcomes

The prognosis and long-term outcomes of EoGD need to be further investigated through additional cohort studies. A recent retrospective cohort study investigated whether esophageal involvement or the anatomic location of eosinophilic infiltration affects the natural course of EGIDs [49]. Among 97 children and adults, 43% had esophageal involvement, which was associated with longer diagnostic delays, more dysphagia, a greater need for chronic therapy, and more progressive disease [49]. Continuous disease was most common in EoG (78%), while progressive and relapsing courses were more frequent in eosinophilic enteritis and colitis. Children and those with single-organ involvement often had continuous disease, whereas adults showed more relapsing or progressive disease [49].

Quinn et al. [50] further examined the natural history and complications in 151 children with gastric or duodenal eosinophilia. Among these children, 25% experienced persistent or recurrent eosinophilia, which was associated with higher EGREFS and duodenal endoscopic abnormalities [50]. The study found that 18% of the patients developed complications, such as anemia, gastrointestinal bleeding, and bowel perforation, most of which occurred years after the initial diagnosis. Importantly, patients presenting with complications at diagnosis or severe endoscopic abnormalities were at a higher risk of future complications [50]. These findings suggest that children with EoGD require close monitoring for disease progression, especially if they present with significant endoscopic abnormalities at the time of diagnosis [50].

## Conclusions

EoGD is a relatively rare but increasingly recognized form of EGIDs with significant clinical implications, particularly in pediatric patients. A multidisciplinary approach, incorporating the expertise of gastroenterologists, allergists, dietitians, and surgeons, is crucial to ensure optimal patient care and management. Specifically, early referral to a gastroenterologist is critical, especially when patients present with persistent gastrointestinal signs and symptoms that do not improve with treatment for common conditions like IBS.

*CD*: celiac disease; *EGD*: esophagogastroduodenoscopy; *HPF*: high-power field; *IBD*: Inflammatory bowel disease; *IgE*: immunoglobulin E; *PUD*: peptic ulcer disease.

## Data Availability

Not applicable.

## Abbreviations

ADCC: Antibody-dependent cell-mediated cytotoxicity
CBC: Complete Blood Count
CCL11: Eotaxin-1
CCL26: Eotaxin-3
ECP: Eosinophil Cationic Protein
ED: Elimination Diet
EDN: Eosinophil-Derived Neurotoxins
EGD: Esophagogastroduodenoscopy
EGIDs: Eosinophilic Gastrointestinal Diseases
EGREFS: Eosinophilic Gastritis Endoscopic Reference Score
EoC: Eosinophilic Colitis
EoD: Eosinophilic Duodenitis
EoE: Eosinophilic Esophagitis
EoG: Eosinophilic Gastritis
EoGD: Eosinophilic Gastroduodenitis
EoI: Eosinophilic Ileitis
EoJ: Eosinophilic Jejunitis
EoN: Eosinophilic Enteritis
GI: Gastrointestinal Tract
hpf: High-Power Field
Hr-QoL: Health-Related Quality of Life
IBD: Inflammatory bowel disease
IBS: Irritable Bowel Syndrome
IL-5Rα: IL-5 receptor alpha
ILC2s: Innate Lymphoid Cells
NICU: Neonatal Intensive Care Unit
Th2: T Helper 2
